# Remarkable Case of Tubercular Excavation Communicating with the External Air through an Aperture between the Ribs

**Published:** 1830-10-01

**Authors:** 


### XXXVIII.

### Remarkable Case of Tubercular Excavation communicating with the External Air through an Aperture between the Ribs.

Mr. Macklin, æt. 47.—His symptoms at present, July 25, 1830, are frequent, small, troublesome cough—expectoration of yellow, puriform sputa, occasionally tinged with blood, and rather abundant—pain at times in right side of chest and shoulder — decubitus on *left* side only, with head rather high—aspect phthisical—disposition to perspire at nights—pulse feeble—appetite good.

Chest narrow, contracted—on deep inspiration left side only expands—right shoulder droops. Immediately below the right sterno-clavicular articulation, but rather nearer the shoulder, the integuments for a space of two inches, or rather more, in diameter, are reddish and very thin. On coughing or expiration, they swell out into a tumour nearly as large as a goose egg, which almost seems ready to burst. The integuments are drawn inward on inspiration. Great part of the sternal end of first rib, and right side of upper bone of sternum between first and second rib are absorbed, and the space between the first and second rib is consequently enlarged from above downwards. The integuments here and the neighbouring bones are very tender on pressure.

*Percussion and Auscultation.* Whole of *left* thorax sounding tolerably sonorous. Respiration in this side unequal, but generally puerile—no râle, nor pectoriloquy.

Lower part of *right* thorax anteriorly, and right back dull upon percussion. The respiration in this side imperfect every where, but especially so below, although almost cavernous above; no pectoriloquy heard, but gargouillement on coughing, at the apex of the lung.

*History.* Three years ago was in good health, but suffered from a cough the preceding Winter. In October 1827, his wife died of confirmed phthisis, which had lasted for two years. In the succeeding February, he caught a cold, for which he treated himself at first, but without success. Mr. Cosgreave was called in and prescribed for him. Cough, with more or less expectoration remained, and in the Winter of 1827-8 he went to Madeira, where he gained flesh surprisingly, but never lost his cough. In the Spring of 1828 he returned, and from that time till a month or so ago he still continued to suffer from the cough and expectoration, with occasional pain in the side, and emaciation. Three weeks or a month ago emphysema of the right side of the neck, and parts contiguous to the tumour, took place; it subsided in a few days. After the subsidence of the emphysema, the tumour below the right clavicle was discovered by Dr. Johnson. Within the last three weeks the integuments have grown thinner.

*Plaister and roller to defend the thin integuments from bursting—sedatives—moderately good diet.*

26th. Awoke last night and found that the integuments had given way, without any particular exertion of which he was aware. Suffers little aggravation of the symptoms in consequence. Discharges air through the aperture at each expiration.

*Light compress.*

*Aug. 1st.* A considerable quantity of purulent discharge issues from the wound on coughing—expectorates by the mouth little else than glairy mucus (probably from bronchi and other lung)—feels inconvenience if the dressings obstruct in any way the egress of matters from the wound—cough troublesome.

*Pil of Morph. Acet. and Conium, ter die—ale, meat, &c.*

*Monday, 9th.* We examined the patient. The expectoration by coughing, from the aperture between the ribs, was very considerable, and attended with a loud discharge of wind, so much resembling a common cough, that it was difficult to say whether the noise was made by the mouth or the intercostal aperture. Very little expectoration now comes from the mouth, except when in the horizontal position, when a small proportion is discharged in the common way. The cough is still more troublesome than before the new opening took place—he wastes in flesh—and the total discharge is increased. He has, however, no fever; and he sleeps a little by the aid of Battley's sedative. He takes sulphate of quinine in acidulated infusion of roses.

22*d*. The patient has daily lost ground; and there has lately come on a colliquative diarrhœa, which opiates and absorbents cannot check. The expectoration from the external wound continues profuse, and the rush of air, at each expiration, and especially when coughing, is considerably greater than that which is emitted by the trachea and larynx. The constant discharge from the wound now renders his life a burthen to him, and death is become acceptable. On the 24th of August, he expired. The body was examined by Dr. Dill and Dr. Johnson. The following is Dr. Dill's report.

Inspection. The body was much emaciated—the skin covering the sternal ends of the second and third ribs of the right side was ulcerated, and a fistulous opening, which communicated with the right thoracic cavity, was formed in the intercostal space between them. On removing the sternum and cartilaginous portion of the ribs, it was found that this fistulous aperture terminated in an immense cavern within the right lung, which was capable of containing at least one pint and a half. This cavity was lined with a tolerably dense membrane, which adhered both anteriorly and behind to the ribs, and, although in some parts there intervened between this sac and the costal pleura a small quantity of pulmonary parenchyma, which had not been as yet destroyed, in general no remains of lung could be perceived beyond the sac, which was in immediate union with the pleura which lined the chest. The destructive process, which had formed this enormous cavity within the substance of the lung, had extended anteriorly through a portion of the sac and costal pleura to the ribs, the sternal ends of the first two of which it had rendered carious, and the third and fourth were so far diseased as to break with ease when but slightly pressed upon. Across the lower half of this tubercular cavern ran, in an oblique direction, a small shred of lung, which appeared to consist principally of the pleura, which had lined the sulcus between the superior and middle lobes; and towards the left side of the floor a fistulous opening was discovered, which admitted the extremity of the little finger. By slitting down the division of the trachea which is devoted to the right lung, the third branch, which this tube subdivides into, was found to terminate in this opening; and in its immediate neighbourhood was seated another aperture, which was likewise the termination of another branch. It was principally through these openings that the matter, which was expectorated by the mouth, made its way into the trachea, and these free outlets, aided by the external aperture upon the surface of the chest, had furnished such a ready exit to the contents of the cavity that, although almost all the upper and middle lobes were destroyed, there was scarcely any tubercular fluid in the sac. The lower lobe was also extensively diseased and contained several smaller cavities, which freely communicated with that now described. One of these cavities, large enough to contain a pigeon's egg, in place of being ragged, uneven, and suppurating like the rest, was lined with a fine, smooth, mucous membrane. This cavity had been obviously the seat of previous disease, which Nature had arrested by forming this artificial membrane; but as the remainder of the lung—even that in the immediate neighbourhood of this healed vomica—was irremediably disorganized, this sanative effort could have given but a trifling, if any check to the progress of the symptoms. The right lung was in a tolerably healthy state, having only a few hard tubercles imbedded in its upper lobe, and the heart exhibited no manifestation of disease.*

* The immense magnitude of the excavation in this case, and the quantity of matter which it must have always contained before it burst externally, occasioned the absence of pectoriloquy. Those acquainted with auscultation will perceive that the indications supplied by percussion and auscultation were remarkably correct. The case is, on the whole, a rare and curious one. The patient was under the care of the editor, but other avocations prevented his attending with regularity and minuteness to the symptoms, which will account for the concise and abbreviated form of the notes.

